# Reconstructing the major skull myology of the giant stem‐amphibian *Mastodonsaurus*: A case study for early tetrapods

**DOI:** 10.1111/joa.70239

**Published:** 2026-09-14

**Authors:** Daniel Schwarz, Stephan Lautenschlager, Florian Witzmann, Raphael Moreno, Eudald Mujal, Rainer R. Schoch

**Affiliations:** ^1^ Department of Palaeontology State Museum of Natural History Stuttgart (SMNS) Stuttgart Germany; ^2^ Institute of Zoology and Evolutionary Research Friedrich Schiller University Jena (FSU) Jena Germany; ^3^ School of Geography, Earth and Environmental Sciences University of Birmingham Birmingham UK; ^4^ The Lapworth Museum of Geology University of Birmingham Birmingham UK; ^5^ Museum für Naturkunde Leibniz Institute for Evolution and Biodiversity Science Berlin Germany; ^6^ Institut Català de Paleontologia Miquel Crusafont (ICP‐CERCA) Universitat Autònoma de Barcelona, Edifici ICTA‐ICP Cerdanyola del Vallès Catalonia Spain; ^7^ Department of Paleontology, Institute for Biology University of Hohenheim Stuttgart Germany

**Keywords:** early tetrapods, evolutionary morphology, extant phylogenetic bracket, functional anatomy, soft‐tissue reconstruction, temnospondyls

## Abstract

The Devonian fish‐to‐tetrapod transition and the subsequent conquest of land involved multiple amphibious stem and early tetrapod lineages, yet how these animals reduced their dependence on aquatic environments remains uncertain. Feeding is central to this transition, as early tetrapods likely moved from predominantly aquatic suction feeding to jaw‐based prey prehension and processing. Although recent functional approaches—including morphometrics, functional morphology and computational modelling—offer new perspectives, their interpretive strength depends on realistic soft‐tissue inputs, particularly cranial musculature. Here, we reconstruct the principal cranio‐mandibular (skull) musculature of the Middle Triassic capitosaur temnospondyl *Mastodonsaurus giganteus* to provide an explicit anatomical foundation for future functional and modelling studies of stem and early tetrapods and to refine ecological interpretations for this species. We formulate alternative muscle hypotheses within an extant phylogenetic bracket (EPB) framework that explicitly reflects uncertainty in temnospondyl placement (temnospondyl versus lepospondyl origin of lissamphibians). We developed hypotheses from a literature review and digital dissections of contrast‐enhanced soft‐tissue μCT datasets from selected extant bracket taxa and outgroups and assessed them against osteological correlates in *M. giganteus*. Our reconstruction indicates a more differentiated adductor system than commonly assumed, with subdivision of the adductor mandibulae externus and internus complexes and an inferred intramandibular component consistent with a cartilaginous sesamoid (‘*cartilago transiliens*’) functioning as a force‐transmitting pulley. We further provide osteological evidence for previously unrecognised pterygoideus components, suggesting additional jaw‐closing capacity and a potential contribution to rapid gape formation. The ‘tongue’ in *M. giganteus* was likely comparatively weak and less flexibly movable, while as a stereospondyl, it possessed a comparatively strong neck and specialised anterior ribs. Collectively, these traits are consistent with a mainly crocodile‐like bite‐and‐hold prehension ecology and likely indicate kinetic‐inertial feeding. Our muscle reconstruction further suggests a more balanced jaw‐opening/closing system that may have enabled compensatory suction during gape formation.

## Graphical abstract

We reconstruct the major cranial musculature of the giant stem amphibian *Mastodonsaurus giganteus*, revealing a differentiated jaw‐adductor system, a probable sesamoid pulley, and previously unrecognised muscles. This EPB‐based case study establishes an anatomical framework for modelling feeding in early tetrapods and supports interpretations of capitosaur bite‐and‐hold prehension, kinetic–inertial feeding, and compensatory suction.

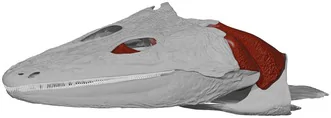

## 1 INTRODUCTION

The fish‐to‐tetrapod transition during the Middle to Late Devonian and the subsequent conquest of land (Clack, 2012; Long & Gordon, 2004; Shubin, 2008) likely occurred in multiple amphibious stem‐ and early tetrapod lineages. Many of these stem and early tetrapods exhibit traits that may have enabled semi‐terrestrial lifestyles or, at least, brief terrestrial excursions (Ahlberg, 2018; Benton, 2014; Clack, 2012; Schoch, 2014a). However, how these animals ultimately reduced their dependence on aquatic environments remains uncertain. A key but understudied aspect of the water‐to‐land transition concerns the feeding system in stem and early tetrapods (Heiss et al., 2018; Schwarz et al., 2023). These animals likely shifted from suction feeding, the most prevalent and probably ancestral aquatic prey‐capture strategy, to more terrestrial prey‐capture modes such as mandibular grasping and jaw prehension (Heiss et al., 2018; Neenan et al., 2014; Reilly & Lauder, 1990; Schwarz et al., 2023). By contrast, stem and early tetrapods likely retained the ancestral vertebrate mode of oropharyngeal food processing, reconstructed as involving biting or arcuate chewing (Schwarz et al., 2023, 2026). How these behaviours functioned across aquatic and terrestrial environments, which differ fundamentally in their physical properties, remains uncertain and depends strongly on the functional morphology of the feeding apparatus, including its musculature.

While early work speculated on and discussed the feeding behaviour and diet of stem and early tetrapods (Olson, 1961; Romer, 1947; Schaeffer & Rosen, 1961; Watson, 1926), subsequent quantitative research was initially driven largely by extant comparative models. Stem and early tetrapods were often treated only superficially, and key interpretations were derived primarily from these model‐based approaches (Bramble & Wake, 1985; Heiss et al., 2018; Reilly, 1996; Reilly & Lauder, 1990; Schwarz et al., 2023). Although these studies have established important links between morphology and function, they cannot represent the full range of conditions present in the highly diverse stem and early tetrapods. Recent studies have therefore adopted additional approaches, including morphometrics or morphospace analysis with functional or evolutionary inference (Deakin et al., 2024; Rawson et al., 2022), functional morphological approaches (Lemberg et al., 2021; Markey & Marshall, 2007) or computational modelling (Fortuny et al., 2011, 2016, 2017; Lautenschlager et al., 2016). These novel methods can provide additional perspectives and enable new interpretations. However, they are prone to incorrect or oversimplified conclusions if not considered in light of comparative evidence from extant models (Schwarz et al., 2023).

A key next step, therefore, is to apply these increasingly quantitative and model‐based approaches to focal fossil clades that (i) capture the morphological disparity of stem and early tetrapods and (ii) occupy pivotal positions in the water‐to‐land transition, while explicitly grounding functional inferences in expectations derived from extant comparative frameworks. Among the most informative candidates for such integrative analyses are temnospondyls, whose diversity and long history of phylogenetic scrutiny make them an ideal test case for linking cranial form, function and feeding behaviour in deep time. Early work on temnospondyl feeding was largely deductive, relying on broad morphological comparisons with extant taxa (Damiani et al., 2009; Howie, 1970; Sulej & Majer, 2005). More recently, such qualitative inference has increasingly been extended by more detailed anatomical considerations (Witzmann & Schoch, 2013; Witzmann & Werneburg, 2017) or complemented/replaced by computational approaches, including finite element analysis (FEA) (Fortuny et al., 2011, 2017; Lautenschlager et al., 2016). However, the outputs of such models are highly sensitive to their input assumptions. To date, these inputs have largely been limited to cranial reconstructions, with little consideration of the mandible. In addition to accurate surface geometry, they require estimates of key muscular parameters, including the overall number, attachment sites, orientation, cross‐sectional area/volume and force‐generating capacity of the muscles. Consequently, robust reconstructions of skull myology in extinct species are essential for providing reliable input for subsequent computational modelling.

**FIGURE 1:**
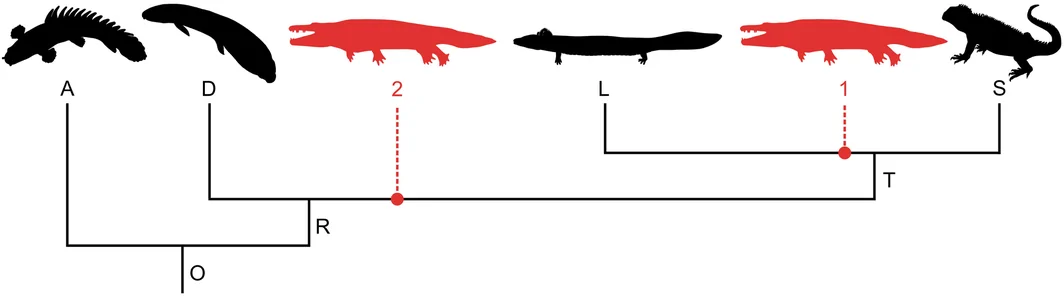
Phylogenetic position of temnospondyls according to different hypotheses and the resulting extant phylogenetic bracketing scenarios. **A**, actinopterygians; **D**, dipnoi; **L**, lissamphibians; **O**, osteichthyans; **R**, Rhipidistia (or Dipnotetrapodomorpha, which contains Tetrapodomorpha plus Dipnomorpha); **S**, sauropsids; **T**, tetrapods. The red additions to the cladogram highlight the association of temnospondyls according to either **1** the ‘temnospondyl theory’ or **2** the ‘lepospondyl theory’ of the evolution of amphibians. The temnospondyl theory renders dipnoans as the outgroup, while the lepospondyl theory assigns this role to actinopterygians.

The question of where to place temnospondyls is crucial for reconstructing traits related to their soft tissues, as their placement affects the phylogenetic bracket (Figure 1) and thus potentially the resulting conclusions about their cranial myology (muscular anatomy) (Witmer, 1998). The first attempts to apply Hennig's phylogenetic systematics placed temnospondyls as stem‐amphibians and thus as early crown tetrapods (‘temnospondyl hypothesis’) (Milner, 1988, 1993). Subsequent work has proposed alternative hypotheses for lissamphibian origins, including diphyly/polyphyly (frogs derived from temnospondyls, with salamanders and caecilians derived from lepospondyls) (Carroll, 2007), and a lepospondyl origin for lissamphibians (‘lepospondyl hypothesis’) (Laurin & Reisz, 1999; Marjanović & Laurin, 2019). More recent combined‐evidence studies that incorporate extensive molecular constraints generally favour lissamphibian monophyly and show reduced support for diphyly/polyphyly (Hime et al., 2021; Kligman et al., 2023). Consequently, most recent analyses tend to support the ‘temnospondyl hypothesis’ (Kligman et al., 2023; Ruta et al., 2003; Schoch, 2014a; Sigurdsen & Green, 2011). Nevertheless, because both the ‘temnospondyl hypothesis’ and the ‘lepospondyl theory’ remain debated, we consider both and discuss the corresponding alternative muscle‐reconstruction scenarios.

Under the ‘temnospondyl hypothesis’, the closest extant phylogenetic bracket (EPB) for temnospondyls, as stem‐amphibians, would encompass lissamphibians on the one side and sauropsids on the other side. By contrast, under the ‘lepospondyl theory’ of the rise of amphibians, the closest EPB for temnospondyls, as stem tetrapods, would comprise crown tetrapods, such as lissamphibians and amniotes on the one side, and extant early branching sarcopterygians such as lungfish and potentially coelacanths on the other side.

Both hypotheses agree that lissamphibians may make up one side of the phylogenetic bracket or at least part of it. Lissamphibians, especially salamanders, are widely regarded as useful extant models to investigate the behaviour and anatomy of early tetrapods, including temnospondyls (Carroll, 2007; Carroll & Holmes, 1980; Reilly et al., 2006; Schoch et al., 2021; Schwarz et al., 2023). Other extant amphibian model taxa may be more suitable for specific functional anatomical comparisons. For example, the greatly expanded pectoral girdle of temnospondyls may make frog musculature informative for studies of axial functional anatomy. Here, however, we focus on the skull's functional anatomy, which is arguably most conserved among salamanders (Schwarz et al., 2023). However, in previous studies, salamander model species seem to have been chosen largely ad hoc, driven by availability, broad lifestyle similarity or external resemblance. Given the substantial diversity of salamanders in ecology, muscular anatomy and developmental biology (Bonett, 2021; Rose, 2003), incorporating ontogenetic anatomical change and the effects of heterochrony may strengthen this model‐organism framework and improve the selection of species suited to particular research questions.

Many salamanders exhibit a biphasic life history, developing from aquatic larvae into sexually mature adults. However, adult phenotypes range from metamorphic forms, which are often more terrestrial, to paedomorphic forms that typically retain larval features and remain predominantly aquatic. Paedomorphosis, as a heterochronic shift in development, can affect not only ecology (aquatic vs. terrestrial habits) but also cranial osteology and myology (Kleinteich et al., 2014; Lauder & Reilly, 1990; Rose, 2003; Schwarz, Konow, Porro, & Heiss, 2020; Ziermann & Diogo, 2013). Larval and neotenic morphotypes often show more complex hyobranchial systems and more differentiated cranial muscles (Heiss & Grell, 2019; Ziermann, 2019). They typically have more numerous/differentiated adductor and depressor mandibulae heads, which may merge or get lost during development/metamorphosis. By contrast, some hyobranchial muscles may become more pronounced after metamorphosis with the development of tongues for terrestrial feeding (Lauder & Reilly, 1990; Schwarz, Konow, Porro, & Heiss, 2020).

By contrast, temnospondyls did not undergo a ‘true’ metamorphosis comparable to that of extant lissamphibians (Schoch & Witzmann, 2024). Instead, most temnospondyls underwent a slow and steady acquisition of adult features, and some even show evidence of direct development (Schoch, 2009; Schoch et al., 2024; Schoch & Witzmann, 2024; Witzmann, 2006). However, certain temnospondyls appear to have evolved an increased developmental speed later in ontogeny (Schoch, 2010; Schoch & Fröbisch, 2006; Schoch & Witzmann, 2024). This apparent shift from the gradual acquisition of adult traits to a more distinctly ‘metamorphic’ developmental mode seems to align with arguments supporting the temnospondyl hypothesis. Regardless, given these findings, it seems most crucial to select suitable heterochronic examples for use in EPB to reconstruct a fossil species' myology. Overall, the temnospondyl anatomical development appears to partially resemble that of salamanders up to a certain neotenic stage (Schoch & Witzmann, 2024). This similarity is especially evident in the bulky, complex hyobranchial system with multiple ceratobranchials (Witzmann, 2013; Witzmann & Schoch, 2013). Conversely, salamanders also show an open cheek region (maxilla ending as a free process without a bony cheek/temporal arch), resembling the early osteological condition of (certain) temnospondyls such as various branchiosaurids (Carroll, 2007; Schoch & Carroll, 2003; Schoch & Milner, 2008). This creates a paradox: The skulls of metamorphic salamanders more strongly resemble the early developmental cranial conditions of temnospondyls (Schoch, 2014b), whereas the hyobranchial systems of larval and paedomorphic salamanders resemble the adult temnospondyl conditions (Witzmann, 2013). A preliminary literature review of extant phylogenetic bracketing taxa suggests that the paedomorphic salamander myology is less derived (Allis Jr, 1922; Carroll & Holmes, 1980; Holliday & Witmer, 2007; Ziermann et al., 2019) and may therefore be more suitable for reconstructing temnospondyl conditions.

Against this background, we reconstructed skull musculature to enable more nuanced functional and ecological interpretation of early tetrapods and fossil taxa more broadly. We present a reconstruction of the major cranial and mandibular myology of the Middle Triassic capitosaur temnospondyl *Mastodonsaurus giganteus*. As a case study, we integrate and evaluate explicitly formulated alternative hypotheses within an extant phylogenetic bracket (EPB) framework. We developed these hypotheses from a targeted literature review and detailed digital dissections of contrast‐enhanced soft‐tissue μCT datasets for carefully selected extant bracket taxa and relevant outgroups. This study specifically aims to: (1) establish a transferable EPB‐based framework for skull myology that can be applied in future comparative studies of vertebrate head musculature; (2) re‐evaluate and, where justified, revise hypotheses concerning the presence and configuration of temnospondyl jaw muscles; (3) use the resulting reconstruction to refine inferences about capitosaur feeding ecology and functional capabilities; and (4) provide a transparent anatomical basis for future modelling of temnospondyl feeding behaviour, particularly prey capture and food processing.

## 2 MATERIALS AND METHODS

### 2.1 Myological terminology

The terminology of the cranial myology, especially that of the mandibular adductor muscles, varies widely according to authors and schools of thought, in addition to changes that have occurred over time; see, for example, (Diogo & Abdala, 2010; Edgeworth, 1935; Lakjer, 1926; Luther, 1914; Ziermann et al., 2019). Due to its relatively broad usage and simplicity, we adopt the terminology developed by Luther (1914) and Lakjer (1926), which Carroll and Holmes (1980) explain in detail.

### 2.2 Selection of study taxa and specimens

We chose the capitosaur *Mastodonsaurus giganteus* from the Middle Triassic of Germany as a temnospondyl case‐study taxon. The large, roughly crocodile‐shaped *M. giganteus* likely occupied an apex predatory role in freshwater habitats (Schoch et al., 2024). Skull lengths reached approximately 1.2 m, implying a total body length of about 5 m (Schoch, 1999). Anatomical and depositional evidence indicates that it was predominantly aquatic and suggests an opportunistic feeding strategy, likely emphasising primarily piscivory (Schoch et al., 2024). *M. giganteus* was chosen because of the availability of often well‐preserved specimens recovered from rich and diverse Fossillagerstätten (Mujal et al., 2025; Schoch et al., 2022; Schoch & Seegis, 2016). Due to their availability, we studied over 40 *M. giganteus* specimens, including a fully articulated skull (SMNS 54677), various isolated but well‐preserved hemimandibles and crania, as well as fragmentary remains (Table 1). Additionally, *M. giganteus*, as the largest known temnospondyl (Schoch, 1999), commonly features large, well‐ossified skulls, enhancing the visibility of otherwise faint or extremely small muscle attachment sites that would likely be difficult to detect in smaller species.

The general and ontogenetic or heterochronic criteria discussed in the introduction suggest that the closest EPB candidates on the lissamphibian or salamander side for studying soft tissue are early branching, relatively large, neotenic, and primarily aquatic taxa. From this perspective, cryptobranchids, given their exceptionally large body size and basal position in salamander phylogeny, may initially seem particularly well suited. However, they actually display a mixed array of both larval and more metamorphic traits, while also showcasing a potentially derived lifestyle adapted to a bottom‐dwelling among rocks in fast‐flowing, well‐oxygenated rivers and streams (Browne et al., 2014; Deban & Wake, 2000; Ishikawa et al., 2022; Matsumoto et al., 2024).

The ancestral amphibian lifestyle was likely primarily semi‐aquatic, comprising an aquatic larval stage in lentic freshwater habitats (e.g. ponds and small lakes) and a more terrestrial adult stage that nonetheless remained dependent on water (Schoch, 2014a). In broad terms, the ancestral condition can be characterised as a moist‐skinned, carnivorous tetrapod closely associated with (fresh)water habitats, in which feeding, reproduction and many activities occurred in or around water (Liedtke et al., 2022). Hence, we argue that the more phylogenetically derived *Necturus maculosus*, whose lifestyle (Eycleshymer, 1906; Harris Jr, 1959) may be more reminiscent of the early ontogenetic phase of the ancestral amphibian state, is a more suitable candidate. Although *Necturus maculosus* differs from the inferred ancestral amphibian lifestyle in being fully aquatic, this condition may better approximate capitosaur ecology, given that capitosaurs were also likely strongly tied to aquatic environments (Schoch, 1999). To extend the candidate‐perspective from the lissamphibian side, we argue that adding *Siren lacertina*, as an extant, early branching giant (up to ~1 m) with a ‘very early larval‐morphotype’ (Martof, 1973; Schwarz et al., 2021, 2023), may be beneficial for identifying potential differences in ontogenetic muscular development.

The EPB to study the condition under the temnospondyl hypothesis requires an opposing amniote candidate. The phylogenetically best suited may be the Tuatara (*Sphenodon punctatus*). Yet we argue that adding a crocodilian representative that is more morphologically and ecologically similar, such as the American alligator (*Alligator mississippiensis*), may yield a more nuanced picture. *Neoceratodus forsteri* (Dipnoi) and *Polypterus senegalus* (Actinopterygii) were further added as outgroup taxa. We selected *P. senegalus* as the earliest‐branching extant actinopterygian with a closed skull roof. Together, these features provide an outgroup baseline that may more closely approximate temnospondyl conditions than salamanders, which have open skull roofs.

Table 1 summarises the specimens examined in this study, listing their voucher locations and, where relevant, their μCT scanning parameters and MorphoSource (https://www.morphosource.org/) identification numbers.

**TABLE 1:** Specimens anatomically studied for myological inference.

Group	Studied species	Specimen ID and repository	MorphoSourceID	μCT scanning details
Lissamphibians	*Necturus maculosus*	**SMNS** z‐herp 16547.3	000897560	**VetCore** Skull region (adult), DECT (I_2_E) XRadia MicroXCT‐400 (80 kV, 100 μA) 23.3 μm voxel size
	*Siren lacertina*	**PMJ** Amph 387	000363163	**TPW** Skull region (adult), DiceCT (I_2_E) GE Phoenix Nanotom® m (140 kV, 400 μA) 22.5 μm voxel size
Reptilians	*Sphenodon punctatus*	**FMNH**: Amphibians and Reptiles:11115	000779927	**UF Nanoscale** Skull region (adult), DiceCT GE Phoenix v|tome|x m 240 (160 kV, 220 μA) 42.9 μm voxel size
	*Alligator mississippiensis*	**OUVC**:11415	000072948	**OUMF** Skull region (subadult), in vivo GE eXplore Locus (in vivo) (120 kV, 32 μA) 49.3 μm voxel size
Dipnoi	*Neoceratodus forsteri*	**ANU**:73578	000383355	**CTLab** Skull region (juvenile), DiceCT (I_2_E) In‐house assembled custom μCT Scanner 16.5 μm voxel size
Actinopterygian	*Polypterus senegalus*	**UMMZ**:FISH:195008	000483259	**MZRMC** Skull region (adult), DiceCT Nikon Metrology XT H225 ST (100 kV, 200 μA) 17.3 μm voxel size
Temnospondyl	*Mastodonsaurus giganteus*	**Complete skulls: SMNS** 54677 [μCT], 80884 **Isolated well‐preserved crania: SMNS** 4698, 4706, 4974, 54675 [photogrammetry], 54678, 54679, 80878, 80913, 81966, 97115, 97220, 97221 **(hemi)mandibles: SMNS** 56634, 80871, 80872, 80874, 80879, 80881, 80882, 80883, 80884, 80913, 81310, 81376, 82013, 83322, 97222, 97223 **and fragmentary remains: SMNS** 56643, 80875, 80878, 80890a/c, 80891, 80892, 80893, 80894, 80895, 80897, 80952, 81038, 81040, 81041, 81368, 97115, 97224 **Clavicle: SMNS** 81301 **Interclavicle: SMNS** 81297	000898899	**MFN3DL** Skull (adult) YXLON FF85 CT (275 kV, 300 μA) 120 μm voxel size

*Note*: **Institutions**: **ANU**, Australian National University (Collection of Rocks, Minerals, and Fossils, Research School of Earth Sciences); **CTLab**, National Laboratory for X‐ray Micro Computed Tomography of the Australian National University; **FMNH**, Field Museum of Natural History [Chicago] (Zoology: amphibian and reptile collection); **MFN3DL**, Museum für Naturkunde Berlin 3D‐Laboratory; **MZRMC**, Museum of Zoology Research Museums Center; **OUMF**, Ohio University MicroCT Facility; **OUVC**, Ohio University Vertebrate Collection; **PMJ**, Phyletisches Museum Jena; **SMNS**, State Museum of Natural History Stuttgart; **TPW**, TPW Prüfzentrum GmbH; **UF Nanoscale**, UF Nanoscale Research Facility of the University of Florida; **UMMZ**, University of Michigan Museum of Zoology, Research Museums Center; **VetCore**, VetCore Facility [VetImaging] of the University of Veterinary Medicine Vienna.

Abbreviations: DECT, dual energy μCT, (DiceCT) diffusible iodine‐based contrast‐enhanced computed tomography, I_2_E, alcoholic iodine staining.

### 2.3 Anatomical (μCT) analysis of extant species

To conduct the EPB analysis, we utilised both our own and publicly available high‐resolution micro‐CT scans from MorphoSource, which included the heads of the selected species (Table 1). The reconstruction of skull musculature for the selected EPB species was based on a combination of a literature review and digital dissection of these μCT scans. Detailed μCT scan settings are in Table 1 and the respective MorphoSource records. *Siren lacertina* scanning protocols are described in detail in Schwarz et al. (2021).

We examined the major cranial muscles through digital dissection (segmentation) of μCT datasets. By contrast, pectoro‐cranial and neck muscles (protractor pectoralis, cleidomastoideus, and trapezius) were largely assessed from the literature because the available μCT scans did not consistently include sufficient postcranial anatomy. We carried out segmentation using Avizo 3D 2021.2 (Thermo Fisher Scientific). Muscles were manually segmented from the tomographic data, while bones were segmented using a combination of manual and semi‐automatic thresholding methods, allowing for comprehensive three‐dimensional anatomical assessment. The segmented muscles were then exported as surface files (.obj) and imported into Blender—a 3D modelling and rendering package (Version 4.3.0), Blender Foundation (https://www.blender.org/), for visual analysis under the guidance of published data on myology of the taxa in question. We applied this approach to all taxa except *Sphenodon punctatus*, whose myology has already been described in sufficient detail (Holliday & Witmer, 2007; Jones et al., 2009) and used the μCT data solely to confirm previous findings. Blender surface models of the resulting 3D reconstructions for *Polypterus senegalus*, *Neoceratodus fosteri*, *Necturus maculosus*, *Siren lacertina* and *Alligator mississippiensis* are provided in the supplementary materials S2–S6.

### 2.4 *Mastodonsaurus giganteus*: 3D Skull model generation and muscle reconstruction

A fully articulated skull of *M. giganteus* (SMNS 54677) was μCT‐scanned at the Museum für Naturkunde Berlin (MfN) using a Comet Yxlon system (Comet YXLON GmbH, Hamburg, Germany; Equipment identification ID RRID:SCR_020917). The volume dataset was segmented in Avizo 3D to isolate individual cranial elements and separate them from internal sediment; minor taphonomic cracks were repaired at this stage. The segmentation was exported as a triangulated surface mesh for further processing in Blender.

In Blender, the better‐preserved side was chosen as the primary template and missing or damaged structures were restored by duplicating and transferring corresponding elements from the opposite side, where available. The restored half was mirrored to generate a bilaterally repaired model. Because automated merging routines produced errors due to the large mesh size, the two halves were manually aligned and stitched. The tabular horns and parts of the occipital region, which are incompletely preserved on both sides of SMNS 54677, were therefore supplemented using a photogrammetric 3D model (created in Agisoft Metashape v.1.8.3, professional license, educational version, https://www.agisoft.com; edited in MeshLab v.2020.07, https://www.meshlab.net) of an additional specimen (SMNS 54675). Well‐preserved exemplary teeth were reconstructed by a combination of segmentation and manual modelling/sculpturing and placed along the marginal and palatal tooth rows, because complete tooth segmentation was hindered by low contrast and small tooth size in parts of the dataset. The larger fangs were segmented where present and multiplied to be added where absent, following the morphology and positional pattern observed in the available material.

Muscles were modelled in Blender using the add‐on MyoGeneratorRemix (Miguel Díaz de León; https://github.com/MiguelDLM/MyoGeneratorRemix), which implements and extends the MyoGenerator workflow described by Herbst et al. (2022). Following the procedure of Herbst et al. (2022), we first defined origin and insertion areas for each muscle inferred from the EPB on the digital cranium and mandible models. Each remaining muscle was then generated as an individual 3D object, and its volume was manually sculpted to fit the available anatomical space while remaining consistent with the inferred attachment areas. The completed reconstruction was subsequently used to generate anatomical overview renderings from different perspectives.

## 3 RESULTS

### 3.1 Extant phylogenetic bracketing of skull muscles

The resulting muscle pattern from the EPB approach (Table 2) provided a directly comparable, specimen‐based reference for the presence/absence, path, relative size and three‐dimensional spatial relationships of muscles among taxa. These data formed the basis for EPB‐supported hypotheses regarding muscle presence and paths (Witmer, 1995, 1998), which were then used to reconstruct the myology of *Mastodonsaurus giganteus*.

**TABLE 2:**
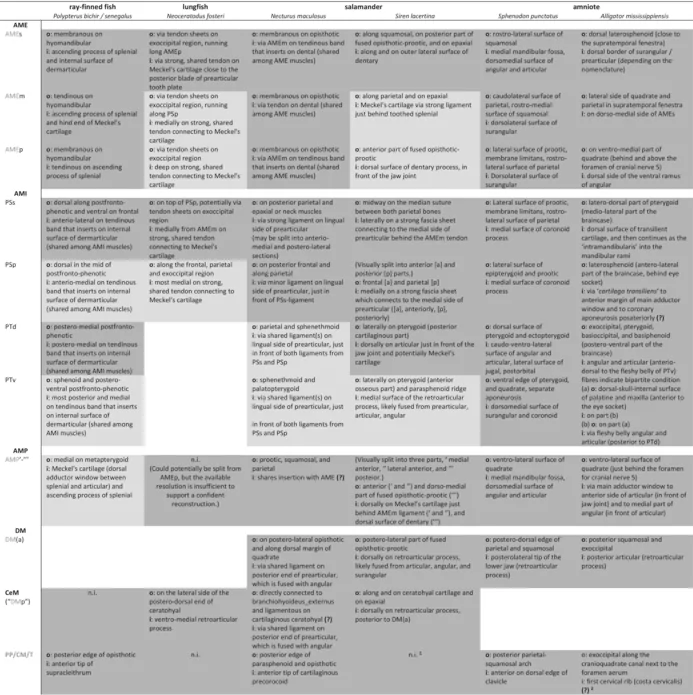
Presence of major mandibular muscles in selected EPB taxa.

*Note*: **(?)** indicates uncertainty regarding the origin, insertion, or general homology of the muscle under consideration. ^**1**^The cleidomastoideus, as a neck muscle, is likely absent in sirenids because of their eel or snake‐like elongated bodies and aquatic lifestyle. ^**2**^ Note that the crocodilian neck musculature is comparatively understudied and remains somewhat enigmatic, partly because the ‘proatlas’ and the loss of the clavicle and interclavicle alter attachment sites for several neck muscles. To assess the comparative presence of cranial muscles, we combined a literature review with detailed anatomical analyses based on digital dissections of μCT datasets. The material examined is listed in Table 1, and the relevant references are: **Salamanders** (Bauer, 1997; Carroll & Holmes, 1980; Harris Jr, 1957; Piatt, 1939, 1940; Schwarz, Konow, Roba, & Heiss, 2020; Schwarz et al., 2021); **Amniotes**: crocodilia (Bona & Desojo, 2011; Holliday et al., 2022; Holliday & Witmer, 2007; Iordansky, 2000, 2010; Tsai & Holliday, 2011) as well as 3D reconstructions from https://sketchfab.com/holliday; as well as *Sphenodon punctatus* (Holliday & Witmer, 2007; Jones et al., 2009); **Dipnoi**, *Neoceratodus forsteri* (Bemis, 1984, 1986; Bemis & Lauder, 1986; Diogo, 2008; Fox, 1965; Ziermann et al., 2018); and **Actinopterygii**: *Polypterus bichir* (Allis Jr, 1922; Lauder, 1980; Traquair, 1870). Findings derived from the literature are highlighted in dark grey, whereas results newly obtained from digital dissections are shown in light grey. For a more detailed understanding and visualisation of the myology of the study taxa, see Supplementary Materials S2–S6.

Abbreviations: **mAME**, (musculus, m.) adductor mandibulae externus (a complex muscle that may consist of); **AMEs**, adductor mandibulae externus superficialis; **AMEm**, adductor mandibulae externus medialis; **AMEp**, adductor mandibulae externus profundus; **AMI**, adductor mandibulae internus (a complex muscle that may consist of up to four parts; PSs, PSp, PTd, and PTv); **AMP(’‐”’)**, adductor mandibulae posterior (sometimes divided into three parts,’‐”’); **CeM**, ceratomandibularis; **CM**, cleidomastoideus; **DM(a/p)** depressor mandibulae (anterior/posterior); **i**, insertion of major mandibular muscles; **n.i**., not identifiable; **o**, origin of major mandibular muscles; **PP**, protractor pectoralis; **PSs**, pseudotemporalis superficialis; **PSp**, pseudotemporalis profundus; **PTd**, pterygoideus dorsalis; **PTv**, pterygoideus ventralis; and **T**, trapezius.

### 3.2 Inferring capitosaur skull muscles via EPB

Under the ‘temnospondyl hypothesis’, our comparative, phylogenetic considerations (EPB approach) suggest the presence of a singular or bipartite DM (DMp potentially absent), a tripartite AME complex (superficialis, medialis and profundus), a tripartite to quadripartite AMI complex (pseudotemporalis superficialis and profundus, as well as a pterygoideus muscle, which may be split into pars dorsalis and ventralis), and a singular AMP and CM constitute the Bauplan of capitosaur temnospondyls such as *M. giganteus*. By contrast, under the ‘lepospondyl hypothesis’, temnospondyls could be argued to exhibit a singular AMP and DM (potentially present as CeM), tripartite AME, and a bipartite AMI, as they are more likely to lack pterygoideus muscles (both pars dorsalis and ventralis).

### 3.3 Validation via fossilised osteological correlates

**FIGURE 2:**
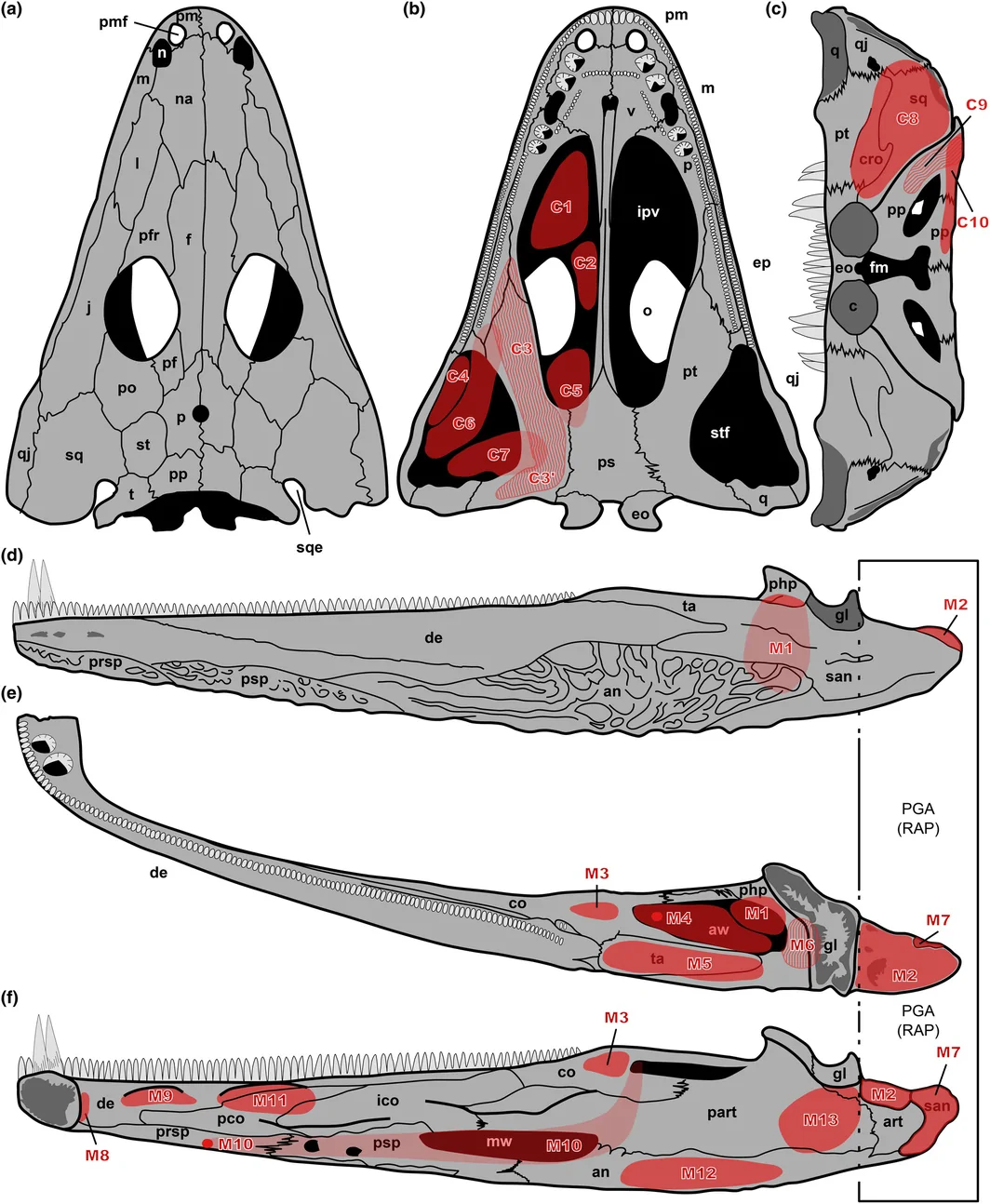
Muscle attachment sites (MAS) in *Mastodonsaurus giganteus*. Views shown are: (a) dorsal cranium; (b) ventral cranium; (c) posterior (‘occipital’) cranium, rotated by 90° to save space; (d) lateral view of the left hemimandible; (e) dorsal view of the left hemimandible; and (f) medial (‘lingual’) view of the left hemimandible, mirrored along the longitudinal axis. Semi‐translucent red regions at 65% opacity highlight identified surface attachments. Where (parts of) MAS lie beneath other bones in the displayed perspective, they are represented in red at 25% opacity (C5‐7, M1 and M10) for increased transparency. MAS that extend to‐ or are located solely on the reverse side of a bone are indicated with a semi‐translucent wave‐dashed pattern (C3 and M6). To avoid excessive visual overlap, this method is applied selectively only to certain MAS in each view. **an**, angular; **art**, articular; **aw**, adductor window; **co**, coronoid; **cro**, crista obliqua; **de**, dentary; **ep**, ectopterygoid; **eo**, exoccipital; **f**, frontal; **fm**, foramen magnum; **gl**, glenoid; **ipv**, interpterygoid vacuity; **ico**, intercoronoid; **j**, jugal; **l**, lacrimal; **m**, maxilla; **mw**, meckelian window; **n**, naris; **na**, nasal; **o**, orbit; **p**, parietal; **part**, prearticular; **pco**, precoronoid; **pf**, postfrontal; **pfr**, prefrontal; **PGA**, post‐glenoid area; **php**, re‐articular (hamate) process; **pm**, premaxilla; **pmf**, premaxillary fenestra; **po**, postorbital; **pp**, postparietal; **prsp**, presplenial; **ps**, parasphenoid; **psp**, postsplenial; **pt**, pterygoid; **q**, quadrate; **qj**, quadratojugal; **RAP**, retroarticular process; **san**, surangular; **sq**, squamosal; **sqe**, squamosal embayment; **st**, supratemporal; **stf**, subtemporal fenestra; **t**, tabular; **ta**, torus arcuatos; and **v**, vomer.

The broad selection of material enabled us to identify a comprehensive view of muscle attachment sites in *M. giganteus*. Figure 2 displays the results as a triplane diagram of a hemimandible and cranium. Additional sample photographs documenting our muscle attachment analyses are provided in the appendix (S1).

The muscle attachment sites identified in *M. giganteus* form a consistent set of osteological correlates that can be mapped onto the cranium and mandible by their principal bony substrates and positions within the skull framework. These correlates underpin the myological reconstruction presented here and were interpreted in light of comparative evidence from our EPB‐based digital dissections. Table 3 summarises the recognised attachment sites and their proposed assignments to the reconstructed muscles under the EPB framework.

**TABLE 3:** Muscular attachment sites in *Mastodonsaurus giganteus* and reconstructed muscles.

Structure	Number	Location on (main)bone(s) of insertion	Type of MAS	Reconstructed muscle
Cranium	C1	Ventral pfr	Weak rugosity	Pseudotemporalis superficialis (PSs)
	C2	Ventral F	Weak rugosity?	Pseudotemporalis profundus (PSp)
	C3	Ventral pt. (anterior)	Fossa with crest	Pterygoideus dorsalis (PTd)
	C3’	Ventral pt. (posterior)	Fossa with crest	Pterygoideus ventralis (PTv)
	C4	Ventral qj	Rugosity	Adductor mandibulae externus superficialis (AMEs)
	C5	Ventral pf + p	Rugosity	Adductor mandibulae externus profundus (AMEp)
	C6	Ventral sq. + j	Rugosity	Adductor mandibulae externus medialis (AMEm)
	C7	Ventral sq. + st	Rugosity	Adductor mandibulae posterior (AMP)
	C8	Posterior sq	Crest	Depressor mandibulae (DM)
	C9	Anterior pp	Fossa with crest	Cleidomastoideus (CM)
	C10	Posterior pp	Fossa with crest	Epaxial (EP*)
Mandible	M1	In aw, lateral part+php	Fossa	Adductor mandibulae externus profundus (AMEp)
	M2	On RAP, dorsal san + art	Fossa with crest	Depressor mandibulae (DM)
	M3	Dorsal co	Rugosity	Pseudotemporalis superficialis (PSs)
	M4	In aw, dorsal an	Tubercle	Adductor mandibulae externus medialis (AMEm)
	M5	Dorsal ta	Fossa with crest	Adductor mandibulae externus superficialis (AMEs)
	M6	In aw, below gl on art	Fossa	Adductor mandibulae posterior (AMP)
	M7	Medial san	Fossa with crest	Pterygoideus dorsalis (PTd)
	M8	Anterior de	Rugosity	Geniohyoid (GH*)
	M9	Medial on de	Fossa, crest & rugosity	Intramandibularis 1 (IM’*)
	M10	Meckel's cartilage	Fenestra	Pseudotemporalis profundus (PSp)
	M11	Posterior de + pco	Fossa, crest & rugosity	Intramandibularis 2 (IM”*)
	M12	Medial an	Fossa and rugosity	Intramandibularis 3 (IM”’*)
	M13	Posterior‐medial part	rugosity	Pterygoideus ventralis (PTv)

*Note*: Bold asterisks* indicate reconstructed muscles that are intramandibular or cranio‐vertebral (neck) muscles. These were reconstructed by inference from their location and relative size and were excluded from the EPB‐based reconstruction, which was restricted to cranio‐mandibular muscles. **Abbreviations** of anatomical structures (for location see Figure 2): **an**, angular; **aw**, adductor window; **co**, coronoid; **de**, dentary; **f**, frontal; **ico**, intercoronoid; **j**, jugal; **p**, parietal; **part**, prearticular; **pco**, precoronoid; **pf**, postfrontal; **pfr**, prefrontal; **pp**, postparietal; **pt**, pterygoid; **qj**, quadratojugal; **san**, surangular; **sq**, squamosal; **RAP**, retroarticular process; **ta**, torus arcuatos. A question mark ‘?’ indicates uncertainty about the presence of a MAS.

Table 4 compares the outcomes of the two EPB scenarios with the muscle reconstructions derived from the observed attachment sites to assess which scenario is better supported by the myological reconstruction.

**TABLE 4:**
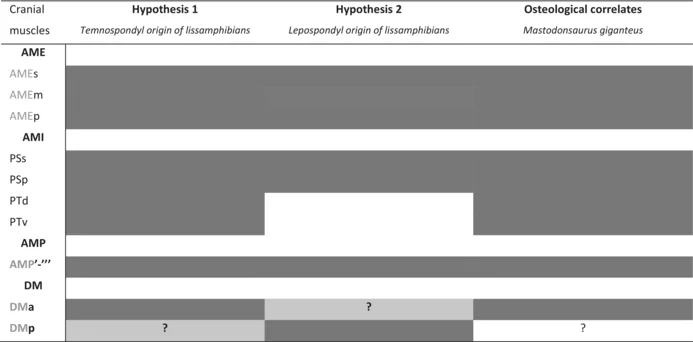
Comparing reconstructed myology with two EPB scenarios.

*Note*: Dark‐grey‐shaded cells indicate the presence of osteological correlates and their respective muscles under the given hypothesis of lissamphibian origin. The question mark and light grey shading denote uncertainty about the presence of the respective muscle due to conflicting EPB information.

### 3.4 Digital reconstruction of skull musculature

**FIGURE 3:**
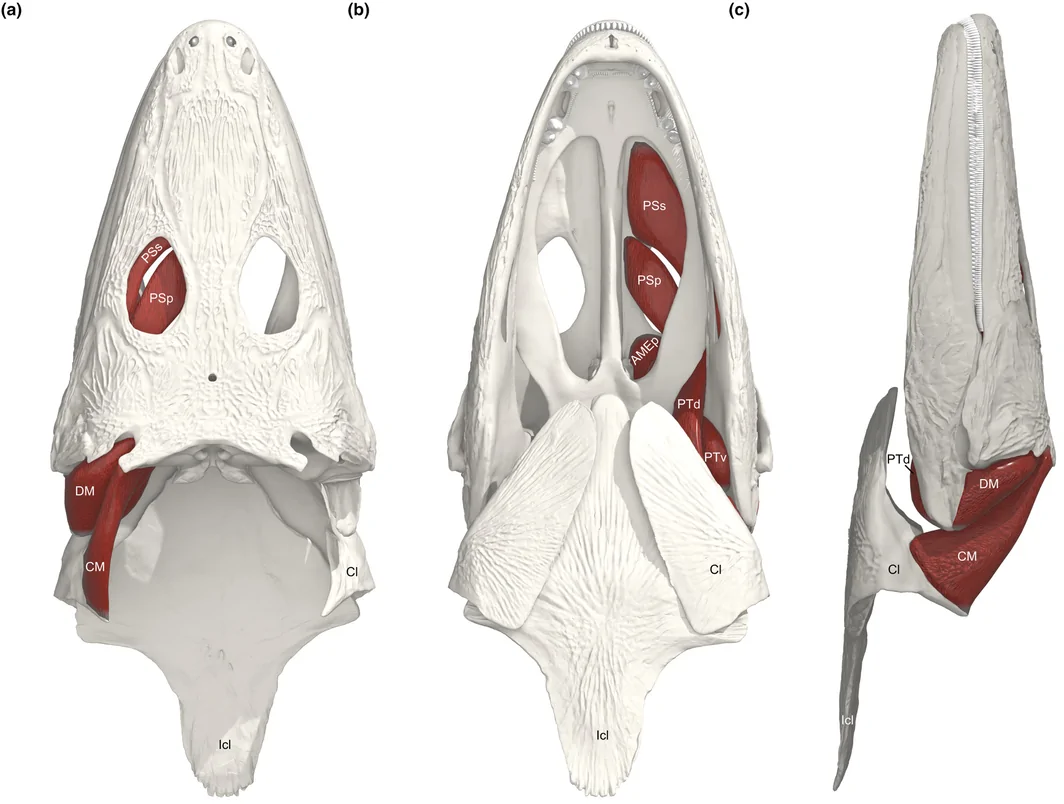
Rendering of the skull, clavicles and reconstructed muscles of *Mastodonsaurus giganteus*. Panels show the skull in (a) dorsal, (b) ventral and (c) lateral views. Most major skull muscles of temnospondyls are hidden within the skull. Externally visible are only the **AMEp**, adductor mandibulae externus profundus; **Cl**, clavicle; **CM**, cleidomastoideus; **DM**, depressor mandibulae; **Icl**, interclavicle; **PSs**, pseudotemporalis superficialis; **PSp**, pseudotemporalis profundus; **PTd**, pterygoideus dorsalis. Teeth are rendered in white and bones in grey to improve visual separation. Note that the intramandibularis ‘‐”’, epaxial and geniohyoideus muscles have not been digitally reconstructed, as their counter attachment sites remain unmodelled.

**FIGURE 4:**
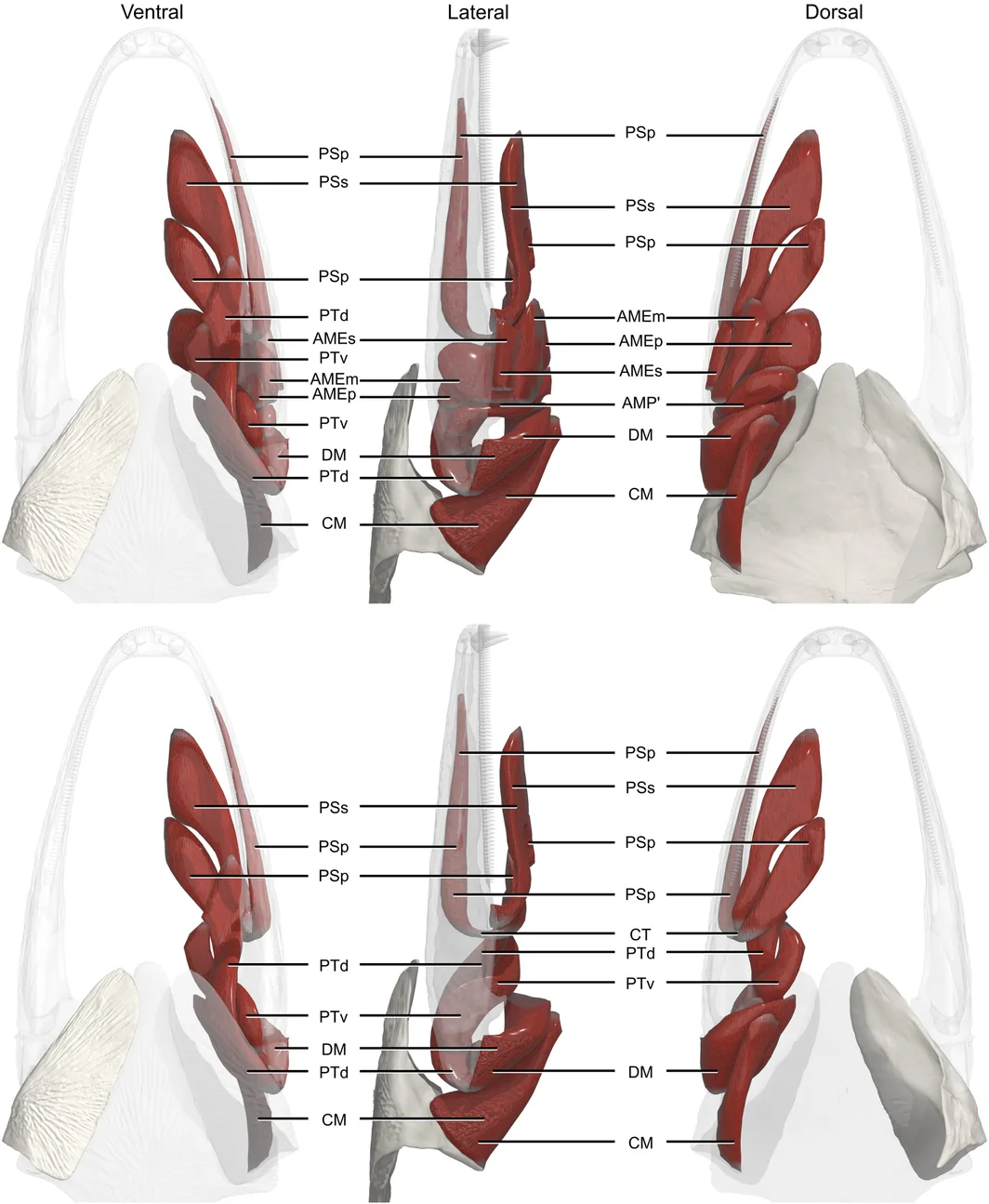
Rendering of the reconstructed muscles of *Mastodonsaurus giganteus*. **AMEm**, adductor mandibulae externus medialis, **AMEp**, adductor mandibulae externus profundus; **AMEs**, adductor mandibulae externus superficialis; **AMP**, adductor mandibulae posterior; **CM**, cleidomastoideus; **DM**, depressor mandibulae; **PSp**, pseudotemporalis profundus; **PSs**, pseudotemporalis superficialis; **PTd**, pterygoideus dorsalis; **PTv**, pterygoideus ventralis. Teeth are rendered in white and bones in grey to improve visual separation. In some views, selected bones and teeth are rendered opaque to facilitate visualisation of muscles located behind them or within parts of the skull.

By integrating EPB‐derived hypotheses with osteological correlates and a 3D reconstruction of the skull of *M. giganteus*, we obtained an internally consistent reconstruction of the major cranial muscles that conforms to the available cranio‐mandibular space and satisfies all attachment site constraints (Figures 3 and 4). The model yields spatially explicit estimates of muscle origins, insertions and three‐dimensional trajectories, constrained by recognisable attachment sites. The adductor complex is reconstructed as distinct components with attachments mapped to discrete cranial and mandibular regions. Reliable reconstruction of three‐dimensional muscle paths and volumes with tools such as MyoGeneratorRemix requires identifiable origins and insertions. We therefore did not model three muscles as volumetric elements because at least one attachment would have been on non‐ossified soft tissue and could not be localised confidently: intramandibularis 1–3, epaxial musculature and geniohyoideus. We therefore retain their EPB‐supported presence but treat their courses as unresolved.

## 4 DISCUSSION

Using a combination of extant phylogenetic bracketing (EPB) via detailed anatomical considerations from soft tissue contrast‐enhanced μCT scans and literature and comparative considerations of the actual muscle attachment sites in *Mastodonsaurus giganteus* specimens, the most likely myology of capitosaur temnospondyls has been reconstructed.

### 4.1 Functional anatomy of capitosaur skulls

The results of this study suggest that capitosaurs like *Mastodonsaurus*, likely exhibited a myological setup that fits the reconstruction based on the ‘temnospondyl hypothesis’. Thus, in turn, apparently further supporting the currently favoured hypothesis of the amphibian origin of temnospondyls. However, one might argue that *Neoceratodus* should be precluded from EPB due to its peculiar heterochronic development and peculiar lifestyle, which result in an altered morphology when compared with the outgroup genus *Polypterus*, which exhibits all but a depressor mandibulae (DM) muscle. This could suggest that *Polypterus* is the better‐suited candidate for the EPB. Acknowledging that the DM arose from the levator hyoideus (Edgeworth, 1935) and that this switch was likely connected to the rise of more terrestrial lifestyles among Dipnotetrapodomorpha (Rhipidistia), one could argue that the presence of a DM was very likely among temnospondyls. Consequently, in this outgroup scenario, the reconstruction of the mandibular myology of capitosaurs would be identical to that reconstructed under the ‘temnospondyl hypothesis’.

Regardless, this study suggests a general revision of the previously established muscle reconstruction pattern (Fortuny et al., 2017; Konietzko‐Meier et al., 2018; Lautenschlager et al., 2016; Witzmann & Schoch, 2013; Witzmann & Werneburg, 2017). Whereas AME and AMI have often previously been treated as broad, undifferentiated groups, we here subdivide them into their constituent muscles: the AME complex (superficialis, medialis and profundus) and the AMI complex, interpreted as tripartite or quadripartite (pseudotemporalis superficialis and profundus, and pterygoideus dorsalis and ventralis). This study not only provides a more detailed separation of the individual muscles but also suggests a more complex and diverse orientation of these muscles and, hence, their function.

The most striking points might be argued to be connected to the novel, detailed reconstruction of adductor mandibulae externus (AME) and adductor mandibulae internus (AMI). The reconstructed origin and insertion suggest the presence of an intramandibularis portion of pseudotemporalis profundus within the AMI complex. This arrangement would have produced a reflected, approximately U‐shaped musculotendinous course through the anterior corner or angle of the adductor fenestra, wrapping around the pterygoids. At the vertex of this reflection, where the muscle portions converged, a cartilaginous sesamoid may have acted as a pulley, or ‘hypomochlion’, redirecting the line of action and transmitting tensile force while reducing local friction and wear. Such an arrangement would be mechanically consistent with forceful jaw adduction and sustained prey retention, enabling powerful bite‐and‐hold jaw action. Such cartilaginous sesamoids are known from crocodylians and certain turtles and are often referred to as ‘*cartilago transiliens’* (Barghusen, 1960; Holliday & Witmer, 2007; Iordansky, 2000; Schumacher, 1973) and have been argued to have been present in *Parotosuchus helgolandicus* (Witzmann & Werneburg, 2017). The orientation and overall morphology of the pseudotemporalis (superficialis and profundus) suggest that it generated peak force at large gape angles, enabling rapid and powerful closure of the elongate jaws of capitosaurs. Further, this interpretation also supports an explanation for the exceptionally large interpterygoid vacuities in capitosaurs such as *Mastodonsaurus*, as proposed by Witzmann and Werneburg (2017).

The absence of fossilised hyobranchial elements in *Mastodonsaurus* and many other capitosaurs indicates a non‐ossified, comparatively weak hyobranchial system and therefore a ‘tongue’ that was likely unsuitable for generating strong water currents or contact‐based intraoral transport of larger prey. Together with our myological findings, this suggests that capitosaur temnospondyls, as predominantly aquatic and relatively long‐snouted (apex) predators, relied on rapid, powerful biting driven largely by the AMI. Thus, supporting the previously formed hypothesis that certain stem and early tetrapods may have relied on kinetic‐inertial (KI) feeding behaviours (Olson, 1961; Schwarz et al., 2023). In KI feeding, snapping jaws rapidly seize prey, after which rapid head, neck and body movements accelerate it towards the oesophagus. When the jaws open to release the prey, the head slows abruptly or reverses direction, allowing prey inertia to carry it deeper into the oral cavity. This process may occur in a single event or be repeated, with the jaws re‐grasping the prey at progressively more posterior positions until it reaches the pharynx for swallowing (Bels et al., 2023; Gans, 1969; Schwenk, 2000a, 2000b; Schwenk & Rubega, 2005). The mechanism may have resembled the neck‐powered mode of KI feeding, in which neck extension produces dorsal cranial elevation and propels food upwards and backwards along an arc‐like trajectory (Bels et al., 2023; Montuelle et al., 2009; Schwenk, 2000b; Taylor, 1987). Alternatively, it may have resembled a ballistic‐transport‐like behaviour (Bels et al., 2023), in which rapid anterior head movement primarily drives intraoral prey transport. Interestingly, this mode is also observed in certain salamanders (Larsen & Guthrie, 1975; Schwarz et al., 2023). They may have employed either transport mechanism depending on the feeding environment. A ballistic‐style mode driven by rapid anterior head movements may have been advantageous during predominantly aquatic, bottom‐associated feeding. By contrast, arc‐like head‐lifting movements that propelled food backwards may have helped handle larger prey when feeding across the waterline.

A further line of evidence for KI feeding concerns the vertebral anatomy of stereospondyl capitosaurs such as *Mastodonsaurus*. In many stereospondyls, such as *Mastodonsaurus*, the disk‐like intercentrum constitutes the major component of the vertebral centrum, in contrast to the ancestral ‘rhachitomous’ condition, in which the centrum comprises a horseshoe‐shaped intercentrum and paired pleurocentra (Moulton, 1974; Panchen, 1967, 1977; Shishkin, 1989; Warren & Snell, 1991). The enlargement of the intercentrum among various stereospondyls is thought to have reinforced the vertebral column by providing additional rigidity (Carter et al., 2021; Panchen, 1967; Rockwell et al., 1938). This, in turn, likely provided stability against tension and compression forces generated by rapid (KI) movements of the large head and heavy prey in stereospondyls. Additionally, the broad ribs of the anterior trunk bear blade‐like and hook‐like processes, sometimes inaccurately termed ‘uncinate processes’ (Schoch, 1999). These processes likely provided substantial attachment sites for a comparatively strong neck musculature, contributing to both overall stability and powerful head and neck movements. Although this configuration likely reduced flexibility between successive vertebrae, a relatively stiff and reinforced anterior vertebral column could have been advantageous for a large‐headed animal performing rapid head lifting, shaking, striking and inertial prey transport. Regardless, the complex jaw adductor system likely allowed a variety of feeding behaviours, including prey seizure, retention and potential processing.

The main muscular novelty might be the presence of a pterygoideus muscle (pars ventralis and dorsalis). These muscles have not previously been demonstrated in temnospondyls. However, their attachment/origin sites are some of the most clearly visible, featuring not only apparent rugose scars but also striking crests and grooves (see Supplementary Material S1). Their previous oversight may be connected to the fact that their cranial attachment (on the hidden dorsal side of the pterygoids) is not visible from the outside in complete, articulated skulls. However, in μCT scans and broken specimens, this attachment site is clearly visible as a big, roughly L‐shaped concavity or depression (fossa). The presence of these pterygoid muscles not only adds additional bite force for presumably very strong KI‐bites but also seems to have a second function. The general orientation and origin and insertion of the pterygoideus ventralis suggest that muscle contraction may have helped keep the mouth tightly shut when closed or nearly closed; yet when the mouth was opened to a certain degree, its adduction would have resulted in additional, relatively rapid gape formation.

The bipartite or tripartite morphology of the AMP, which was found among certain salamanders, does not seem to represent the ancestral or capitosaur condition, as this condition is not widespread (Carroll & Holmes, 1980) and could not be confirmed in our anatomical considerations based on μCT scans of selected species. Similarly, as argued before, the bipartite condition of the DM, even though present in our selected extant salamander bracket species, is not universal among salamanders and in fact connected to the development of the posterior portion from the levator hyoideus (Diogo & Abdala, 2010; Edgeworth, 1935). Previous work has further shown that the anterior and posterior portions of the depressor mandibulae are frequently only partially separated in salamanders and usually fuse during metamorphosis (Kleinteich & Haas, 2011). Diverse states of a bipartite DM have been reported from various salamander taxa (Bauer, 1997), including ambystomatids (Larsen & Guthrie, 1975; Ziermann & Diogo, 2013), cryptobranchoids (Elwood & Cundall, 1994; Heiss et al., 2013; Matsumoto et al., 2024), sirenids (Schwarz, Konow, Roba, & Heiss, 2020, Schwarz et al., 2021), amphiumids (Erdman & Cundall, 1984), salamandrids (Özeti & Wake, 1969) and plethodontids (Eaton, 1957). A clearly separated (bipartite) DM is usually connected to heterochronic developmental shifts that freeze myological development during the early developmental stages of a merged bipartite DM (anterior and posterior portions). Although a bipartite DM is likely to have been present in *M. giganteus*, this could not be confirmed owing to the comparatively poor preservation of its posterior skull region. Nevertheless, separate reconstruction of the two portions would probably have limited functional implications, as they typically merge or occur in proximity and share a largely common origin and insertion.

In any case, while there might be an argument to be made about additional muscles that might have been present in capitosaurs like *Mastodonsaurus* (DM” or AMP”‐”’), this study identifies a more complex skull myology than previously reconstructed.

In addition to the main cranio‐mandibular muscles, we reconstructed a muscle connecting the clavicle and the posterior cranium using additional surface models of the clavicles. ‘True neck muscles’ (e.g. trapezius and sternocleidomastoideus) are generally considered to have evolved within amniotes from the sarcopterygian protractor pectoralis (cucullaris sensu Edgeworth [1935]) (Diogo & Abdala, 2010; Ericsson et al., 2013; Ziermann et al., 2019). However, in *Mastodonsaurus* and other temnospondyls, a primary role in protracting the pectoral girdle appears unlikely given their gross functional anatomy with massive pectoral girdles and legs. We therefore follow previous authors in referring to the reconstructed muscle as ‘cleidomastoideus’ (Howie, 1970; Sulej & Majer, 2005; Watson, 1958).

In lungfish, the function of the protractor pectoralis has not been studied in detail, but it appears to contribute to suction feeding. The ceratohyal is connected posteriorly to the cranium near the jaw joint (see Supplementary Material S3, 3D reconstruction of *Neoceratodus forsteri*). Anteriorly, the ceratohyal connects to the clavicle via the rectus cervicis, which in turn connects to the posterior cranium via the protractor pectoralis but also links to the general hypaxial musculature (Diogo & Abdala, 2010; Gartner et al., 2022; Jorgensen & Joss, 2016; Ziermann et al., 2019). Although hypaxial muscles and the rectus cervicis generate most suction‐feeding power (Gartner et al., 2022), the protractor pectoralis likely stabilises clavicular motion by anchoring the clavicle to the cranium, acting as a hinge point during ceratohyal rotation. Clavicular movement may therefore reflect the combined action of the protractor pectoralis and hypaxial musculature, coordinated with the rectus cervicis to drive ceratohyal excursions.

In capitosaurs such as *Mastodonsaurus*, the clavicle is markedly more massive and further stabilised by a robust interclavicle, and its connection to the legs precludes substantial movement of the pectoral girdle. Nevertheless, the clavicle bears prominent and large MAS consistent with a homologous muscle, likely capable of generating considerable force. If this force could not protract or rotate the pectoral girdle, it is most plausibly interpreted as contributing to cranial motion. Accordingly, our results support previous suggestions that this muscle may be better termed cleidomastoideus, as it appears to function as a neck muscle similar to the sternocleidomastoideus in amniotes. A strong cleidomastoideus likely helped power cranial elevation, potentially enhancing rapid prey prehension or other ingestion behaviours.

### 4.2 Ecological and evolutionary scenario and implications

While capitosaurs like *Mastodonsaurus*, as stereospondyl temnospondyls have been argued to be able to leave the water for short excursions (Fortuny et al., 2012, 2016; Lautenschlager et al., 2016), either in their pursuit of food, to avoid predators, or during drought periods to find new water bodies to seek shelter and avoid desiccation, they are largely believed to have lived primarily restricted to water (Fortuny et al., 2011; Schoch, 2008). This interpretation is based on many functional anatomical features like their flattened skulls, sometimes reduced ossification and the presence of pronounced lateral line systems that aid the detection of movement under water (Fortuny et al., 2012; Schoch, 2013), and the general body‐plan and limb (micro)anatomy that suggest suboptimal terrestrial locomotion, or potentially inability to support their weight outside of water (Mehmood et al., 2025; Sanchez et al., 2010; Schoch, 2013). This assumption is further corroborated by the fact that their skeletal remains are often found in deposits of former rivers, deltas or lakes (Mujal et al., 2025; Schoch et al., 2022, 2023, 2024; Schoch & Moreno, 2024; Schoch & Seegis, 2016) or seem to showcase heavily ossified bones (including clavicle and interclavicle) that may act as skeletal ballast to keep them on the ground (Konietzko‐Meier et al., 2014) similar as seen in *Metoposaurus* (Kalita et al., 2022).

*Mastodonsaurus giganteus*, the largest known temnospondyl, likely filled a top‐predatory niche in Triassic ecosystems (Schoch et al., 2024). Capitosaurs such as *Mastodonsaurus* are commonly regarded as occupying a niche similar to that of many modern crocodilians due to convergent morphological similarities. These include broadly comparable body form, a parabolic and elongate snout, an akinetic skull and an anterior portion of the AMI that extends far forwards into the suborbital fenestrae of the palate (Lakjer, 1926), likely facilitating rapid, snapping‐like grasping [KI feeding] (Olson, 1961). Accordingly, this functional role of rapidly grasping apex predators within freshwater predator guilds is widely considered to have been taken over later by archosaurs of the crocodile lineage (Fortuny et al., 2011, 2012, 2016; Quarto & Antczak, 2024).

The reconstructed skull muscles of *M. giganteus* are surprisingly balanced when it comes to their task of jaw opening versus jaw closure. The combination of pronounced DM and cleidomastoideus (CM), and the possibly bipartite function of the pterygoideus ventralis (PTv), likely aided comparatively quick and effective mouth opening against the hydrodynamic resistance of the surrounding water. While the DM likely mainly aided the initial ventral rotation of the mandible (‘mandibular depression’), the PTv likely additionally powered quick mandibular depression, while the CM mainly aided the elevation of the cranium (Howie, 1970) to make space for ventral rotation of the mandible, which would otherwise collide with the substrate in ground‐resting longirostrine capitosaurs. The addition of a PTv and the thus altered functional interpretation of potentially relatively fast mouth opening is novel and suggests that capitosaurs like *Mastodonsaurus* potentially used rather quick open‐close‐based grasping behaviours, including stalking‐grasping to ram‐based attacks. This capacity for rapid open‐close‐based grasping further supports KI feeding, including inertial transport of prey.

However, depending on general cranial shape, the potential presence of labial lobes (lateral soft tissue covering of the mouth), which are common among predominantly aquatic amphibians (Deban & Wake, 2000; Heiss & Grell, 2019; Matthes, 1934; Toussaint‐Lardé et al., 2025; Van Wassenbergh & Heiss, 2016), and the size of the preferred prey, compensatory suction feeding may have supported their grasping attacks. Thus, capitosaurs may occupy a more balanced position on the ram–suction continuum than previously thought. In any case, the occurrence of these muscles in both the EPB taxa and our temnospondyl case study suggests that they were probably more widespread among stem and early tetrapods, including other temnospondyls. Given the extreme diversity in cranial shapes among temnospondyls, the pterygoideus could have significantly powered mandibular depression and thus akinetic suction feeding, at least in more brevirostrine taxa, such as metoposaurids or plagiosaurids like *Gerrothorax*, as previously suggested (Witzmann & Schoch, 2013). If present, suction feeding would have differed from the kinetic feeding condition proposed for stem tetrapods such as *Tiktaalik* (Lemberg et al., 2021).

However, detailed functional anatomical studies (e.g. using computational fluid dynamics, CFD) are needed to test whether the forces these muscles could exert were actually enough to power sufficient suction behaviours that could theoretically permit (compensatory) suction, especially for capitosaurs, given their relatively longirostrine, crocodile‐like head shape that might intuitively seem to prevent effective suction feeding.

### 4.3 Limitations, anatomical uncertainties and future directions

Identifying muscle attachment sites (MAS) was often difficult because many are expressed only as small rugose areas that are not visible under all lighting conditions. Moreover, even relatively prominent MAS were not consistently visible across specimens representing the same anatomical region, apparently reflecting differences in preservation and/or preparation, as well as potentially intraspecific variation. We therefore treated an attachment site as reliable only when it was supported by evidence from at least two specimens and included only such sites in our theoretical MAS matrix.

Several regions were particularly challenging. The cranial MAS of AME, AMP and DM were difficult to assess because this part of the cranium is frequently deformed, cracked, intensely mechanically prepared and/or ‘plastered’ (i.e. supplemented with material during preparation). Although there were indications of attachment space consistent with two separate AMP, the lack of a clear separation prevented a confident reconstruction of two distinct muscles. Similarly, the cranial attachments of AMEs and AMEm were reconstructed as separate, but the relevant MAS are faint. In this sense, it remains possible that both subdivisions converge on a single cranial attachment site. The pseudotemporalis profundus presents further uncertainty: its cranial attachment is not always clear, and the size and extent of its intramandibular component (‘intramandibularis’ sensu Witzmann & Werneburg, 2017) cannot be determined because Meckel's cartilage and its attachment to it are not preserved. Nonetheless, it likely extended far anteriorly and occupied much of the intramandibular cavity, as the large postero‐medial Meckelian foramen suggests a need for pressure equilibration associated with substantial volume change during contraction.

Uncertainty also affects the dorsal pterygoid attachment of the two pterygoideus muscles. Because no distinct separation was evident, we reconstructed a broadly even attachment area. Finally, some muscles inferred in our framework (e.g. m. intramandibularis, epaxial musculature, and m. geniohyoideus) were not digitally reconstructed because at least one attachment would have been to non‐ossified soft‐tissue structures (e.g. via connective tissue or ligamentous connections to other muscles), preventing confident reconstruction of their paths and sizes.

The bipartite condition of the DM, observed in the two studied salamander specimen taxa, is a result of the heterochronic developmental condition in the two chosen salamander species, in which differentiation is truncated very early during development when the DM arises from the levator hyoideus (Diogo & Abdala, 2010). While a bipartite DM likely represents the ancestral condition present in *Mastodonsaurus giganteus*, the posterior part of the skull is usually heavily weathered, broken, or supplemented with other material during preparation, and fossilised hyobranchial elements have not yet been discovered. Therefore, it was impossible to clearly identify two individual muscle attachment sites in the specimens studied. Even if capitosaurs possessed a bipartite DM, reconstructing the anterior and posterior heads separately would probably produce a functionally very similar reconstruction to that of a single DM. Both heads would be expected to originate from the posterior cranial wall (likely including the *crista obliqua*) and possibly from the proximal postero‐dorsal ends of elements of the hyoid arch and to insert in proximity on the retroarticular process or post‐glenoid region of the mandible. An analogous configuration is widely observed in neotenic salamanders; supplementary 3D reconstructions of S4 *Necturus maculosus* and S5 *Siren lacertina* show bipartite DM heads that insert closely on the retroarticular process, yielding a largely functionally and visually unified muscle. We therefore reconstructed the DM of *M. giganteus* as a single unit.

Future work could improve MAS‐based reconstructions in several ways: (1) enhance detection and documentation of subtle attachment correlates using standardised raking‐light photography and/or quantitative 3D surface metrology (and, where feasible, higher‐resolution μCT focused on cortical surfaces); (2) expand and strategically curate fossil samples to minimise preservation and preparation biases by prioritising minimally restored specimens and incorporating explicit restoration/deformation maps and retrodeformation workflows for heavily distorted regions; and (3) address uncertain muscle subdivisions and soft‐tissue‐only attachments through explicit alternative reconstructions and sensitivity analyses. The latter could be supported by broader EPB sampling (including additional extant taxa and developmental stages) and by targeted fossil material that exposes otherwise obscured surfaces (e.g. the dorsal pterygoid) or preserves hyobranchial elements. Nevertheless, fossil‐based work remains constrained by the availability and limited access to well‐preserved material, restricted sample sizes and specimen size, which limit and affect both direct observation and CT‐based approaches. For these reasons, efforts to improve reproducibility and reconstruction quality are particularly valuable.

## AUTHOR CONTRIBUTIONS

Conceptualisation: DS; Data curation: DS; Formal Analysis: DS; Funding acquisition: DS and RRS; Investigation: DS, FW, RM, EM, and RRS; Methodology: DS; Project administration: DS; Resources: DS, SL, and RRS; Supervision: DS, SL, FW, and RRS; Validation: DS, SL, FW, RM, EM, and RRS; Visualisation: DS; Writing—original draft: DS; Writing—review and editing: DS, SL, FW, RM, EM, and RRS.

## DECLARATION ON THE USE OF AI

Certain sections of this manuscript were revised for grammar and style using AI‐based tools, including Grammarly (San Francisco, CA, USA), as well as via a locally hosted version (LibreChat) of ChatGPT (OpenAI, San Francisco, CA, USA). All recommendations generated by these tools were thoroughly assessed for accuracy and appropriateness before being incorporated. The authors accept sole responsibility for the manuscript's intellectual content and interpretations.

## Supporting information


**Data S1:** Sample photographs of muscle attachment sites in *Mastodonsaurus giganteus*.
**Data S2**. *Polypterus senegalus* myological 3D reconstruction (blender file).
**Data S3**. *Neoceratodus fosteri* myological 3D reconstruction (blender file).
**Data S4**. *Necturus maculosus* myological 3D reconstruction (blender file).
**Data S5**. *Siren lacertina* myological 3D reconstruction (blender file).
**Data S6**. *Alligator mississippiensis* myological 3D reconstruction (blender file).

## Data Availability

The primary data underlying this study are provided in the supplementary material of this article and via MorphoSource (see Table 1). Additional raw data are available from the corresponding author upon reasonable request.
